# A multiplexed, targeted mass spectrometry assay of the S100 protein family uncovers the isoform-specific expression in thyroid tumours

**DOI:** 10.1186/s12885-015-1217-x

**Published:** 2015-03-29

**Authors:** Juan Martínez-Aguilar, Roderick Clifton-Bligh, Mark P Molloy

**Affiliations:** 1 Department of Chemistry and Biomolecular Sciences, Macquarie University, Sydney, NSW 2109 Australia; 2 Australian Proteome Analysis Facility, Macquarie University, Sydney, NSW 2109 Australia; 3 Kolling Institute of Medical Research, Royal North Shore Hospital, St Leonards, Sydney, NSW 2065 Australia

**Keywords:** S100 proteins, Selected reaction monitoring, Mass spectrometry, Thyroid cancer, Tumour tissue samples

## Abstract

**Background:**

Mounting evidence demonstrates a causal role for S100 proteins in tumourigenesis and several S100 isoforms have shown utility as biomarkers of several types of cancer. The S100 family is comprised of 21 small isoforms, many of them implicated in important cellular functions such as proliferation, motility and survival. Furthermore, *in vivo* experiments have proven the role of S100 proteins in tumour growth and disease progression, while other studies have shown their prognostic value and involvement in resistance to chemotherapy drugs. Taken together, all these aspects highlight S100 proteins as potential therapeutic targets and as a promising panel of cancer biomarkers. In this work, we have developed a mass spectrometry (MS)-based method for the multiplexed and specific analysis of the entire S100 protein family in tumour tissues and have applied it to investigate the expression of S100 isoforms in the context of thyroid cancer, the main endocrine malignancy.

**Methods:**

Selected Reaction Monitoring (SRM)-MS and stable isotope labelling/label-free analysis were employed to investigate the expression of the 21 S100 protein isoforms in thyroid tissue samples. Specimens included 9 normal thyroid tissues and 27 tumour tissues consisting of 9 follicular adenomas (FA), 8 follicular carcinomas (FTC) and 10 papillary carcinomas (PTC).

**Results:**

The multiplexed and targeted mass spectrometry method led to the detection of eleven S100 protein isoforms across all tissues. Label- and label-free analyses showed the same significant differences and results were confirmed by western blot. S100A6, S100A11 and its putative interaction partner annexin A1 showed the highest overexpression in PTC compared to normal thyroid. S100A13 was also elevated in PTC. Reduced S100A4 expression was observed in FA compared to all other tissues. FA and FTC showed reduction of S100A10 and annexin A2 expression.

**Conclusions:**

Targeted mass spectrometry allows the multiplexed and specific analysis of S100 protein isoforms in tumour tissue specimens. It revealed S100A13 as a novel candidate PTC biomarker. Results show that S100A6, S100A11 and Annexin A1 could help discriminate follicular and papillary tumours. The diagnostic and functional significance of S100A4 and S100A10 reduction in follicular tumours requires further investigation.

**Electronic supplementary material:**

The online version of this article (doi:10.1186/s12885-015-1217-x) contains supplementary material, which is available to authorized users.

## Background

Growing evidence links the S100 protein family with cancer [1,2]. This protein superfamily consists of 21 small isoforms (9-13 kDa), which are characterised by their calcium binding ability and conserved primary and secondary structures (the sequence identity among S100 proteins ranges from 16 to 98% [3]). Besides exhibiting some degree of cell specificity, S100 isoform expression depends on environmental factors to regulate important cellular functions such as proliferation and motility. When secreted from cells, some S100 proteins exert chemotactic and angiogenic activities, thereby further contributing to the tumourigenic process [2,4]. Indeed, *in vivo* experiments have proven the role of several S100 proteins in tumour growth and disease progression [2].

Dysregulation of S100 isoform expression has been observed in numerous cancers [2,5] (see Additional file 1: Table S1). For example, S100A4, S100A6, S100A8, S100A9, S100A11 and S100P have shown altered expression in breast, colorectal, gastric, pancreatic and prostate cancer [6-30]. S100A6, S100A13 and S100B have been found as overexpressed in melanoma [31-33], and likewise other S100 proteins have been identified as biomarkers in cancer of the bladder, lung and oesophagus [34-37]. In many instances, the S100 isoforms have also demonstrated prognostic and predictive value, ultimately representing candidate therapeutic targets. For example, S100A4 elevation in colorectal cancer has been associated with decreased survival rate [6,38] and S100A4 inhibition using regulators of β-catenin signalling such as niclosamide or sulindac has shown reduction of liver metastasis in mouse xenograft models [39,40]; S100A6 overexpression in gastric cancer could serve as independent prognostic predictor associated with poor survival [41] and S100A6 knockdown in gastric cancer cells has been shown to inhibit tumour growth *in vivo* [42]. Another S100 isoform, S100P, has been described as a key factor in the aggressiveness of pancreatic cancer [30] and has recently been reported as a promising target of monoclonal antibody therapy, significantly reducing tumour growth and metastasis in a mouse model [43].

Despite the promising potential of the S100 family as a biomarker panel, there is an absence of studies that have addressed the family-wide expression of S100 protein isoforms in clinical samples [44]. This is a challenging task when relying on traditional affinity-based procedures (e.g. immunohistochemistry) due to potential S100 antigen cross-reactivity and the serial nature of optimising and performing IHC assay. We recently developed a targeted mass spectrometry (MS) method based on Selected Reaction Monitoring (SRM) that enables unequivocal structural evidence for the detection of the entire S100 protein family in cancer cells [45]. Here, we have adapted and extended its application for the study of tumour tissues and demonstrate this application in the context of thyroid cancer, the principal malignancy of the endocrine system. The targeted MS approach allowed us to establish the first global picture of S100 protein expression in the three most common tumours of the thyroid gland: follicular adenoma (FA), follicular thyroid carcinoma (FTC) and papillary thyroid carcinoma (PTC). While the latter is the major type of thyroid cancer, tumours with follicular growth pattern (FA and FTC) are indeterminate by fine-needle aspiration (FNA) biopsy, hence hampering the pre-operative diagnosis of tumour malignancy [46,47]. Our results revealed isoform-specific association of S100 proteins in thyroid tumours implicating S100 isoforms have diagnostic potential.

## Methods

### Thyroid tissue samples

Fresh frozen thyroid tissues (9 normal, 9 FA, 8 minimally invasive FTC and 10 classical PTC) were obtained from the neuroendocrine tumour bank at the Kolling Institute of Medical Research, Sydney. Normal samples were obtained from dissection of non-neoplastic tissue from the contralateral lobe of patients who had undergone thyroidectomy or from patients with benign disease where thyroidectomy was indicated. Clinicopathological features are shown in Table 1. All PTC cases showed lymph node metastasis. The research work was conducted with the approval of the human research ethics committees of the Northern Sydney Local Health District (1303-091 M) and Macquarie University (5201300037).

**Table 1:** **Clinicopathological features of the thyroid tissue samples analysed**

Normal tissues				Follicular adenomas			
	Age	Sex			Age	Sex	Tumour size (mm)
Normal-1	42	F		FA-1	36	M	30
Normal-2	20	F		FA-2	51	F	15
Normal-3	78	F		FA-3	30	F	30
Normal-4	61	M		FA-4	46	F	50
Normal-5	51	M		FA-5	46	M	30
Normal-6	34	F		FA-6	14	F	8
Normal-7	84	M		FA-7	21	M	21
Normal-8	35	M		FA-8	68	F	8
Normal-9	29	F		FA-9	59	F	10
Follicular thyroid carcinomas							
	Age of onset	Sex	Tumour size (mm)	T stage	Capsular invasion	Vascular invasion	
FTC-1	43	F	50	3	no	yes	
FTC-2	37	M	40	3	yes	yes	
FTC-3	82	M	45	3	encapsulated	yes	
FTC-4	16	M	13	1	yes	no	
FTC-5	64	F	40	2	no	yes	
FTC-6	64	F	25		yes (focal)	no	
FTC-7	20	M	110	3	no	yes	
FTC-8	22	M	30	2	yes	yes	
Papillary thyroid carcinomas							
	Age of onset	Sex	Tumour size (mm)	T stage	Vascular invasion	Extra-thyroidal spread	
PTC-1	34	F	18	2	no	no	
PTC-2	24	F	30	2	yes	no	
PTC-3	50	M	6	4	no	yes	
PTC-4	59	M	30	3	yes	yes	
PTC-5	33	F	8	1	no	no	
PTC-6	32	F	55	4	yes	yes	
PTC-7	24	F	45	3	no	no	
PTC-8	33	F	28	3	yes	yes	
PTC-9	49	M	44	3	no	yes	
PTC-10	56	M	55	4	yes	yes	

### Metabolic (SILAC) labelling of TPC1 cells for a spike-in standard

SILAC RPMI 1640 media (Thermo Scientific Pierce) was supplemented with 240 mg/mL of L-arginine‐HCl (^13^C_6_^15^N_4_) and 40 mg/mL of L‐lysine‐2HCl (^13^C_6_^15^N_2_) (Cambridge Isotope Labs), 10% dialysed FBS (Life Technologies) and 1% Penicillin-Streptomycin (Gibco). The medium was sterile filtered through a 0.22 micron filter. TPC1 thyroid cancer cells were cultured for at least eight doublings at 37°C in a humidified atmosphere with 5% CO_2_. Upon reaching 70-90% confluence, cells were washed with phosphate buffered saline (PBS), harvested and stored at -80°C until use. Heavy amino acid incorporation was verified by MS.

### Cell and tumour sample preparation

TPC1 cells and tumour samples were suspended in lysis buffer (20 mM HEPES, 1% sodium deoxycholate (SDOC), 0.1% SDS, 150 mM NaCl, 1 mM EDTA, pH 8) with complete, EDTA-free protease inhibitor cocktail (Roche), phosphatase inhibitor tablet (Thermo Scientific Pierce) and lysed on ice with sonication (three cycles of 20 s pulses). Lysates were centrifuged (12 000 g, 10 min, 4°C) and the supernatants retained. Protein quantitation was performed with a BCA protein assay kit (Thermo Scientific).

40 ug of protein from tissue samples and 20 ug of protein from metabollically labelled TPC1 cells were mixed in triplicate before SDS-PAGE. A ratio 2:1 of sample protein to labelled protein was employed given that previous tests measuring the abundance of candidate reference proteins showed varying levels of actual tumour protein amount in different samples due to the presence of blood proteins*.* Labelled TPC1 cells allowed the quantitative analysis of candidate reference proteins and S100 proteins S100A2, S100A6, S100A10, S100A11 and S100A13. It is not unexpected that a particular cell line does not express detectable levels of all S100 isoforms, therefore additional stable isotope-labelled S100 synthetic peptides were spiked into the samples before tryptic digestion (see below for details). While it is conceivable to perform the entire analysis using stable isotope-labelled peptides from reference proteins and S100 proteins, using the SILAC spike-in represents a less expensive approach that does not rely on prior knowledge of the most appropriate peptide sequence to represent the target analyte.

In addition, label-free MS analysis to quantitate all S100 proteins was also carried out as we previously described [45]. To this end, a reference stock sample was prepared, comprised of 700 ug of protein from TPC1 cells grown in normal RPMI medium and 600 ug of protein pooled from the entire set of normal, FA, FTC and PTC tumour lysates. 40 ug of this mixture was loaded onto a Bis-Tris NuPAGE gel for separation. The set of tissue samples was randomised and prepared in batches of four samples (one from each tissue type as far as possible considering the different sample sizes) plus the reference sample. All samples were loaded on gels in triplicate and processed as described below.

Corresponding aliquots of specimens and reference samples were mixed with NuPAGE LDS sample buffer (Invitrogen) and 50 mM dithiothreitol (DTT), incubated at 70°C for 10 min and loaded onto 4-12% Bis-Tris NuPAGE gels (Invitrogen). Proteins were separated using MES running buffer (200 V, 25 min), fixed into the gel with 50% ethanol/10% acetic acid for 1 h and then stained for 1 h with colloidal Coomassie G-250 (Sigma). Gel bands from three mass regions were excised (7-14 kDa, 14-32 kDa and 32-100 kDa), cut into approximately 1 × 1 mm cubes and destained with 50% ACN in 50 mM ammonium bicarbonate (AmBic, pH 8.0). Gel pieces were dehydrated with 100% ACN, speed-vac dried and incubated in 10 mM DTT/100 mM Ambic at 56°C for 1 h followed by treatment with 55 mM iodoacetamide/100 mM AmBic at RT in the dark for 45 min. Gel pieces were dehydrated again, speed-vac dried, rehydrated with 120 uL of 13 ng/uL trypsin solution (sequencing grade modified trypsin (Promega) in 50 mM AmBic and 0.1% SDOC) and kept on ice for 1 h.

Before tryptic digestion, gel pieces from the region 7-14 kDa were spiked with 2 pmol of ^13^C, ^15^N stable-isotope labelled peptide standards ELPSFLG**K** (S100A4), ALNSIIDVYH**K** (S100A8), NIETIINTFHQYSV**K** (S100A9) and 1 pmol of SFWELIGEAA**K** (S100A14) and AVIVLVENFY**K** (S100A16) (SpikeTides TQL, JPT Peptide Technologies, Germany). This step allowed the inclusion of additional labelled S100 peptides not detected in TPC1 cells according to preliminary tests. All samples were then incubated overnight at 37°C. Peptides were extracted thrice with 0.1% SDOC in ultrasonic waterbath for 10 min and then acidified with 1 uL of formic acid. The precipitate was removed by centrifugation (14000 g, 10 min) and the samples were dried in a speed-vac, ready for LC-SRM-MS.

### LC-SRM-MS

The selection of S100 peptides, transitions and optima MS parameters was based on our previous work [45]. The sequences from candidate reference proteins and putative S100 interaction partners annexin A1 (ANXA1) and annexin A2 (ANXA2) were searched by BLAST and Peptide Atlas to identify suitable unique tryptic peptides with more than six amino acids and free of methionine residues. S100 proteins to be quantitated in the tissue samples were detected on preliminary analyses of each sample using the list of SRM transitions reported in Additional file 1: Table S2. These transitions were monitored and also served to assign the retention times of each peptide for subsequent time-scheduled SRM. According to their localisation on the gels, Table 2 lists the peptide sequences and SRM parameters employed for the analysis of S100 proteins and Additional file 1: Tables S3 and S4 the candidate reference proteins in the thyroid tissues.

**Table 2:** **Targeted S100 peptide sequences (region 7-14 kDa)**

Q1	Q3	Rt	Protein.peptide.transition ion.isotopologue	CE	CEXP
846.45	778.41	31.5	S100A1.ELLQTELSGFLDAQK.y7.light	39.3	13
846.45	865.44	31.5	S100A1.ELLQTELSGFLDAQK.y8.light	39.3	13
846.45	1208.62	31.5	S100A1.ELLQTELSGFLDAQK.y11.light	39.3	13
503.27	763.40	23.5	S100A2.ELPSFVGEK. + 2y7.light	22.7	12
503.27	666.35	23.5	S100A2.ELPSFVGEK. + 2y6.light	30.7	11
503.27	579.31	23.5	S100A2.ELPSFVGEK. + 2y5.light	30.7	9
507.27	771.41	23.5	S100A2.ELPSFVGEK. + 2y7.heavy	22.7	12
507.27	674.36	23.5	S100A2.ELPSFVGEK. + 2y6.heavy	30.7	11
507.27	587.33	23.5	S100A2.ELPSFVGEK. + 2y5.heavy	30.7	9
445.75	648.37	25.5	S100A4.ELPSFLGK. + 2y6.light	21.5	19
445.75	551.32	25.5	S100A4.ELPSFLGK. + 2y5.light	25.5	19
445.75	464.29	25.5	S100A4.ELPSFLGK. + 2y4.light	25.5	16
449.76	656.39	25.5	S100A4.ELPSFLGK. + 2y6.heavy	21.5	19
449.76	559.33	25.5	S100A4.ELPSFLGK. + 2y5.heavy	25.5	19
449.76	472.30	25.5	S100A4.ELPSFLGK. + 2y4.heavy	25.5	16
455.22	693.36	16.0	S100A4.TDEAAFQK. + 2y6.light	21.2	20
455.22	564.31	16.0	S100A4.TDEAAFQK. + 2y5.light	21.2	20
455.22	493.28	16.0	S100A4.TDEAAFQK. + 2y4.light	21.2	14
459.23	701.37	16.0	S100A4.TDEAAFQK. + 2y6.heavy	21.2	20
459.23	572.33	16.0	S100A4.TDEAAFQK. + 2y5.heavy	21.2	20
459.23	501.29	16.0	S100A4.TDEAAFQK. + 2y4.heavy	21.2	14
374.22	618.38	18.5	S100A6.ELTIGSK. + 2y6.light	20.3	13
374.22	505.30	18.5	S100A6.ELTIGSK. + 2y5.light	20.3	13
374.22	404.25	18.5	S100A6.ELTIGSK. + 2y4.light	20.3	13
378.22	626.40	18.5	S100A6.ELTIGSK. + 2y6.heavy	20.3	13
378.22	513.31	18.5	S100A6.ELTIGSK. + 2y5.heavy	20.3	13
378.22	412.26	18.5	S100A6.ELTIGSK. + 2y4.heavy	20.3	13
458.25	802.41	17.5	S100A6.LQDAEIAR. + 2y7.light	25.4	13
458.25	674.35	17.5	S100A6.LQDAEIAR. + 2y6.light	23.4	20
458.25	559.32	17.5	S100A6.LQDAEIAR. + 2y5.light	31.4	17
463.25	812.41	17.5	S100A6.LQDAEIAR. + 2y7.heavy	25.4	13
463.25	684.36	17.5	S100A6.LQDAEIAR. + 2y6.heavy	23.4	20
463.25	569.33	17.5	S100A6.LQDAEIAR. + 2y5.heavy	31.4	17
636.85	774.41	24.5	s100A8.ALNSIIDVYHK.y6.light	31.8	13
636.85	974.53	24.5	s100A8.ALNSIIDVYHK.y8.light	31.8	13
636.85	887.50	24.5	s100A8.ALNSIIDVYHK.y7.light	31.8	13
640.86	782.42	24.5	S100A8.ALNSIIDVYHK.y6.heavy	31.9	13
640.86	982.54	24.5	S100A8.ALNSIIDVYHK.y8.heavy	31.9	13
640.86	895.51	24.5	S100A8.ALNSIIDVYHK.y7.heavy	31.9	13
602.98	790.42	30.0	S100A9.NIETIINTFHQYSVK.y13.light	30.4	13
602.98	725.89	30.0	S100A9.NIETIINTFHQYSVK.y12.light	30.4	13
602.98	618.83	30.0	S100A9.NIETIINTFHQYSVK.y10.light	30.4	13
605.65	794.43	30.0	S100A9.NIETIINTFHQYSVK.y13.heavy	30.5	13
605.65	729.90	30.0	S100A9.NIETIINTFHQYSVK.y12.heavy	30.5	13
605.65	622.84	30.0	S100A9.NIETIINTFHQYSVK.y10.heavy	30.5	13
379.21	642.38	18.0	S100A10.DPLAVDK. + 2y6.light	22.5	19
379.21	545.33	18.0	S100A10.DPLAVDK. + 2y5.light	20.5	17
379.21	432.25	18.0	S100A10.DPLAVDK. + 2y4.light	20.5	17
383.22	650.40	18.0	S100A10.DPLAVDK. + 2y6.heavy	22.5	19
383.22	553.34	18.0	S100A10.DPLAVDK. + 2y5.heavy	20.5	17
383.22	440.26	18.0	S100A10.DPLAVDK. + 2y4.heavy	20.5	17
604.80	932.48	26.0	S100A10.EFPGFLENQK. + 2y8.light	26.6	13
604.80	835.43	26.0	S100A10.EFPGFLENQK. + 2y7.light	34.6	13
604.80	466.75	26.0	S100A10.EFPGFLENQK. + 2y8 + 2.light	26.6	13
608.81	940.50	26.0	S100A10.EFPGFLENQK. + 2y8.heavy	26.6	13
608.81	843.45	26.0	S100A10.EFPGFLENQK. + 2y7.heavy	34.6	13
608.81	470.75	26.0	S100A10.EFPGFLENQK. + 2y8 + 2.heavy	26.6	13
386.20	559.32	18.0	S100A11.DPGVLDR. + 2y5.light	24.2	18
386.20	502.30	18.0	S100A11.DPGVLDR. + 2y4.light	24.2	18
386.20	403.23	18.0	S100A11.DPGVLDR. + 2y3.light	20.2	18
391.21	569.33	18.0	S100A11.DPGVLDR. + 2y5.heavy	24.2	18
391.21	512.31	18.0	S100A11.DPGVLDR. + 2y4.heavy	24.2	18
391.21	413.24	18.0	S100A11.DPGVLDR. + 2y3.heavy	20.2	18
530.75	888.45	20.0	S100A11.DGYNYTLSK. + 2y7.light	24.0	14
530.75	725.38	20.0	S100A11.DGYNYTLSK. + 2y6.light	24.0	12
530.75	611.34	20.0	S100A11.DGYNYTLSK. + 2y5.light	24.0	18
534.76	896.46	20.0	S100A11.DGYNYTLSK. + 2y7.heavy	24.0	14
534.76	733.40	20.0	S100A11.DGYNYTLSK. + 2y6.heavy	24.0	12
534.76	619.35	20.0	S100A11.DGYNYTLSK. + 2y5.heavy	24.0	18
519.76	836.45	22.5	S100A13.DSLSVNEFK. + 2y7.light	23.6	13
519.76	723.37	22.5	S100A13.DSLSVNEFK. + 2y6.light	23.6	20
519.76	636.34	22.5	S100A13.DSLSVNEFK. + 2y5.light	23.6	20
523.77	844.47	22.5	S100A13.DSLSVNEFK. + 2y7.heavy	23.6	13
523.77	731.38	22.5	S100A13.DSLSVNEFK. + 2y6.heavy	23.6	20
523.77	644.35	22.5	S100A13.DSLSVNEFK. + 2y5.heavy	23.6	20
372.24	630.38	20.5	S100A13.LIGELAK. + 2y6.light	19.5	19
372.24	517.30	20.5	S100A13.LIGELAK. + 2y5.light	17.5	15
372.24	460.28	20.5	S100A13.LIGELAK. + 2y4.light	25.5	16
376.24	638.40	20.5	S100A13.LIGELAK. + 2y6.heavy	19.5	19
376.24	525.31	20.5	S100A13.LIGELAK. + 2y5.heavy	17.5	15
376.24	468.29	20.5	S100A13.LIGELAK. + 2y4.heavy	25.5	16
624.31	1047.50	20.0	S100A13.SLDVNQDSELK. + 2y9.light	31.3	16
624.31	932.47	20.0	S100A13.SLDVNQDSELK. + 2y8.light	31.3	16
624.31	833.40	20.0	S100A13.SLDVNQDSELK. + 2y7.light	31.3	14
628.32	1055.51	20.0	S100A13.SLDVNQDSELK. + 2y9.heavy	31.3	16
628.32	940.48	20.0	S100A13.SLDVNQDSELK. + 2y8.heavy	31.3	16
628.32	841.41	20.0	S100A13.SLDVNQDSELK. + 2y7.heavy	31.3	14
625.82	1016.54	31.5	S100A14.SFWELIGEAAK. + 2y9.light	29.0	17
625.82	830.46	31.5	S100A14.SFWELIGEAAK. + 2y8.light	29.0	13
625.82	701.42	31.5	S100A14.SFWELIGEAAK. + 2y7.light	32.0	12
629.83	1024.56	31.5	S100A14.SFWELIGEAAK. + 2y9.heavy	29.0	17
629.83	838.48	31.5	S100A14.SFWELIGEAAK. + 2y8.heavy	29.0	13
629.83	709.43	31.5	S100A14.SFWELIGEAAK. + 2y7.heavy	32.0	12
647.87	912.48	31.0	S100A16.AVIVLVENFYK. + 2y7.light	28.0	14
647.87	799.40	31.0	S100A16.AVIVLVENFYK. + 2y6.light	24.0	13
647.87	700.33	31.0	S100A16.AVIVLVENFYK. + 2y5.light	26.0	12
651.88	920.50	31.0	S100A16.AVIVLVENFYK. + 2y7.heavy	28.0	14
651.88	807.41	31.0	S100A16.AVIVLVENFYK. + 2y6.heavy	24.0	13
651.88	708.34	31.0	S100A16.AVIVLVENFYK. + 2y5.heavy	26.0	12

Rt: retention time (min), CE: collision energy; CEXP: cell exit potential.

Samples were analysed using a Waters nanoACQUITY UPLC system coupled to the QTRAP 5500 mass spectrometer (AB Sciex). Weak (A) and strong (B) elution solvents were 0.1% FA (v/v) in water and 0.1% FA (v/v) in ACN, respectively. Samples were loaded onto a Waters Symmetry C18 trapping column (180 um × 20 mm, 5 um particle size) and peptides were separated on a Waters BEH C18 nano UPLC column (100 um × 100 mm, 1.7 um particle size) at 35°C under constant flow rate of 0.4 uL/min. Different elution gradients were used depending on the regions from the gel: a) 7-14 kDa: 3% solvent B for 1 min, 3-40% B in 30 min and 40-85% B in 2 min, b) 14-32 kDa: 3% solvent B for 1 min, 3-40% B in 60 min and 40-85% B in 2 min, c) 32-100 kDa: 3% solvent B for 1 min, 3-40% B in 110 min and 40-85% B in 2 min.

Peptide samples were reconstituted with a solution of 2% ACN/0.1% TFA (v/v) and 17.8 fmol/uL of ^13^C^15^N stable isotope-labelled peptide ESDTSYVS**L**K from human C-reactive protein (Auspep, Australia), used as LC loading control for label-free experiments. Blank injections using a 30 min gradient were performed between each sample. The lists of peptide sequences in Table 2 and Additional file 1: Tables S3 and S4 were targeted using three transitions per peptide with corresponding time-scheduled SRM methods employing cycle times of 1 s and transition windows of 240 s for peptides shown in Table 2 and Additional file 1: Table S3 and 360 s for those listed in Additional file 1: Table S4. The nano-ESI source was operated in positive mode at 2.5 kV with interface heater temperature of 150°C. Curtain gas was set to 20 psi, declustering potential at 70 V and both Q1/Q3 were set at unit resolution. Collision energies (CE) for candidate reference peptides were calculated based on the equations CE(2+) = 0.036*m/z + 8.857 and CE(3+) = 0.0544*m/z-2.4099, taken from Skyline. Peptide identities were corroborated by SRM-triggered IDA MS/MS and searched in the UniProt human database using Mascot v. 2.3.0.

### Data analysis

The potential for peptide interferences was monitored with AuDIT software [48] and signals marked as problematic were removed. Data were processed with Multiquant 3.0 (AB Sciex) using the MQ4 integration algorithm. Manual inspection of the fragment ion peaks was performed to ensure accurate integration. The respective fragment ion peak areas of each endogenous peptide were summed and divided by the transition areas of the isotopically labelled counterparts. The calculated values of different peptides from the same protein were averaged.

In the case of label-free analyses, co-elution of fragment ions and consistency of retention times were used as a guide for peak identity assignment. Signals with CV less than 20% in transition ratios across samples and replicates were considered to represent interference-free conditions. Summed raw peak areas were first divided by the total peak area of the spiked CRP peptide and then against the peak areas of the same peptides present in a reference pool sample that was prepared in the same batch along with the samples of interest. This was done to compensate for differences in sample preparation and system response as detailed in [45]. Again, the average of different peptides from the same protein was calculated. The normalisation factors previously obtained by isotopic labelling were employed for calculation of relative S100 protein expression, thereby keeping maximum precision in this preceding step and allowing the analysis of S100 proteins not detected in the SILAC spike-in standard or not added as heavy peptides before tryptic digestion.

Identification of the most stably expressed endogenous reference proteins across all the thyroid tissue samples (normal, FA, FTC and PTC) was performed using the geNorm algorithm, developed by Vandesompele *et al*. [49]. The geometric mean of SRM-based values of the identified reference proteins was employed as the normalisation factor to calculate the relative expression of S100 proteins in each sample. Comparison of relative S100 protein expression was carried out by one-way anova and Tukey’s post hoc test (p < 0.05) using GraphPad Prism 5.0 software, which was also used for linear regressions analyses. Area under the curve (AUC) of a receiver operating characteristic (ROC) curve was calculated using the ROCR R package.

### Validation of results by Western Blot

Taking into account the normalisation factors derived from the reference proteins, 10 ug of protein from tissue lysates of normal, FA, FTC and PTC samples were separated on 4-12% Bis-Tris NuPAGE gels and electroblotted onto nitrocellulose (Bio-Rad). The membrane was blocked with 5% skim milk in TBS for 1 h at RT. For S100A4 analysis, the membrane was cut in three sections from around 5-17 kDa, 17-32 kDa and >32 kDa. The sections were incubated overnight at 4°C with a) 5-17 kDa - rabbit polyclonal anti-S100A4 (1:100, AbCam), b) 17-32 kDa - rabbit monoclonal anti-Rab7a (1:500, Cell signaling) and c) rabbit monoclonal anti-β-Tubulin (1:500, Cell signaling). For S100A13 analysis, the membrane was cut in two sections (5-17 kDa and >32 kDa) and incubated overnight with either rabbit monoclonal anti-S100A13 (1:3000, AbCam) or rabbit monoclonal anti-Rab7a (1:500, Cell signaling). The secondary antibody was fluorescence-labelled IRDye 800CW goat anti-rabbit IgG (H + L) (1:10 000 in 5% skim milk in TBST, LI-COR), incubated at RT for 1 h and visualized with the Odyssey infrared imaging system.

## Results

### Identification of stable endogenous reference proteins in thyroid tumours

Because tumour specimens contain naturally variable amounts of blood proteins, and in the case of thyroid specimens, thyroglobulin, a simple protein quantitation assay would be insufficient for normalising loading quantities which is a requirement for accurate quantitation. Therefore, our first task was to identify endogenous reference proteins within thyroid tumours that could be used for loading normalisation. Traditionally, ‘housekeeping genes/proteins’ like GAPDH and β-actin have been used as normalisation loading controls; however, increasing evidence shows that these may not be the best reference proteins as their expression stability is compromised when compared across different groups of samples or disease states [49,50]. We used SRM to investigate the expression stability of 18 proteins/genes commonly reported as internal reference standards based on gene expression profiling studies [50,51] (Additional file 1: Tables S3 and S4).

**Figure 1:**
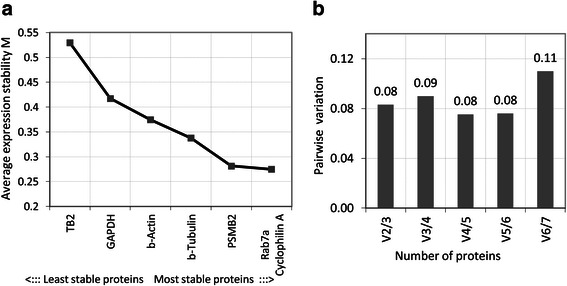
**Determination of reference proteins using the geNorm algorithm. a)** Average expression stability values of candidate reference proteins; **b)** Determination of the optimal number of reference proteins for normalisation. Pairwise variations Vn/n + 1 are calculated between two sequential normalisation factors and stepwise inclusion of the most stable remaining control protein.

In the geNorm method by Vandesompele *et al*. [49], pairwise comparisons between each of the candidate proteins are carried out and an expression stability value M is calculated, which represents the average pairwise variation across all samples of a particular protein compared with the remaining proteins tested. In a first instance, the geNorm algorithm showed proteasome subunit beta type-4 (PROS26) and proteasome subunit beta type-2 (PSMB2) as the protein pair with the highest stability, followed by several ribosomal proteins, valosin containing protein (VCP), Ras-related protein Rab7a and cyclophilin A. β-Tubulin, β-Actin and GAPDH showed higher variation (see Figure 1a). This result served to verify the consistency of our reference protein dataset since PSMB2, PROS26, ribosomal proteins and VCP are all involved in the ubiquitin-proteasome system [52-55]. In order to minimise the possibility of co-regulation we kept proteins belonging to different functional classes: Rab7a (regulator of endo-lysosomal trafficking), cyclophilin A (regulator of protein folding), PSMB2 (proteasome, proteolytic activity), β-Tubulin (cytoskeletal protein), GAPDH (glycolytic protein) and protein TB2 (receptor accessory protein). We retained and tested β-actin, another cytoskeletal protein, given its frequent use in most expression studies.

From the selected subset, Rab7a and cyclophilin A were the most stable proteins identified (Figure 1a) and pairwise variation analysis of the corresponding normalisation factors helped to determine that Rab7a, cyclophilin A and PSMB2 compose a minimal set of stable endogenous reference proteins in thyroid tissues (Figure 1b).

### Detection of S100 protein family by targeted MS

**Figure 2:**
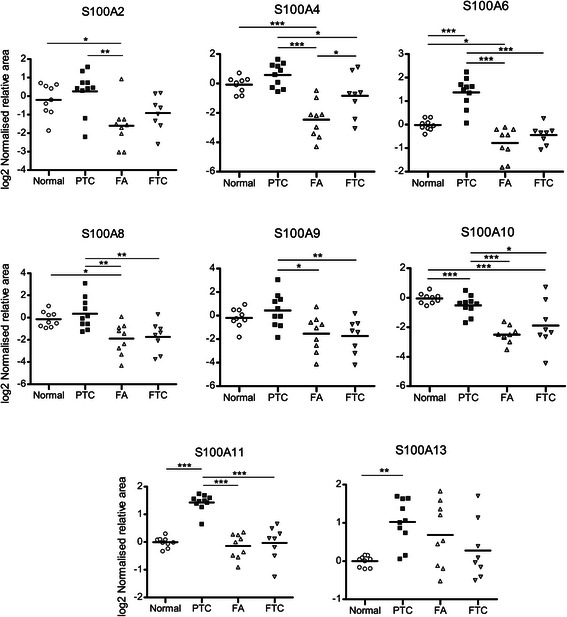
**Differential expression of S100 proteins in normal thyroid and tumour tissues, analysed by spike-in SILAC-SRM.** Normalised expression values were log-transformed and significant changes of S100 protein isoform expression were identified by one-way anova followed by Tukey’s post hoc test (*p < 0.05, **p < 0.01, ***p < 0.001). Results are expressed against the mean S100 protein expression in normal thyroid tissue samples.

Our targeted MS assay for all members of the S100 protein family revealed that the following eleven S100 protein isoforms were detected across all normal, FA, FTC and PTC tissues: S100A1, S100A2, S100A4, S100A6, S100A8, S100A9, S100A10, S100A11, S100A13, S100A14 and S100A16 (Figure 2 and Additional file 1: Figure S1). The MS assay employing the SILAC spike-in standard was highly reproducible with median coefficient of variation (CV) of 5.8%. Although each S100 peptide has its own MS characteristics, our previous work with spiking experiments of six stable isotope-labelled peptides (ELPSFVGE**K**, S100A2; ELPSFLG**K**, S100A4; LQDAEIA**R**, S100A6; DPGVLD**R**, S100A11; LIGELA**K**, S100A13 and AVIVLVENFY**K**, S100A16) demonstrated linear response with up to 3 pmol on-column [45] and we have made broad estimates of the limits of quantitation to be approximately 0.5 fmol (considering lowest concentration with CV <20%), which is consistent with the commonly observed detection limits for peptide SRM being in the sub-femtomolar range [56].

### Targeted MS revealed S100A13 as a novel candidate marker of PTC

**Figure 3:**
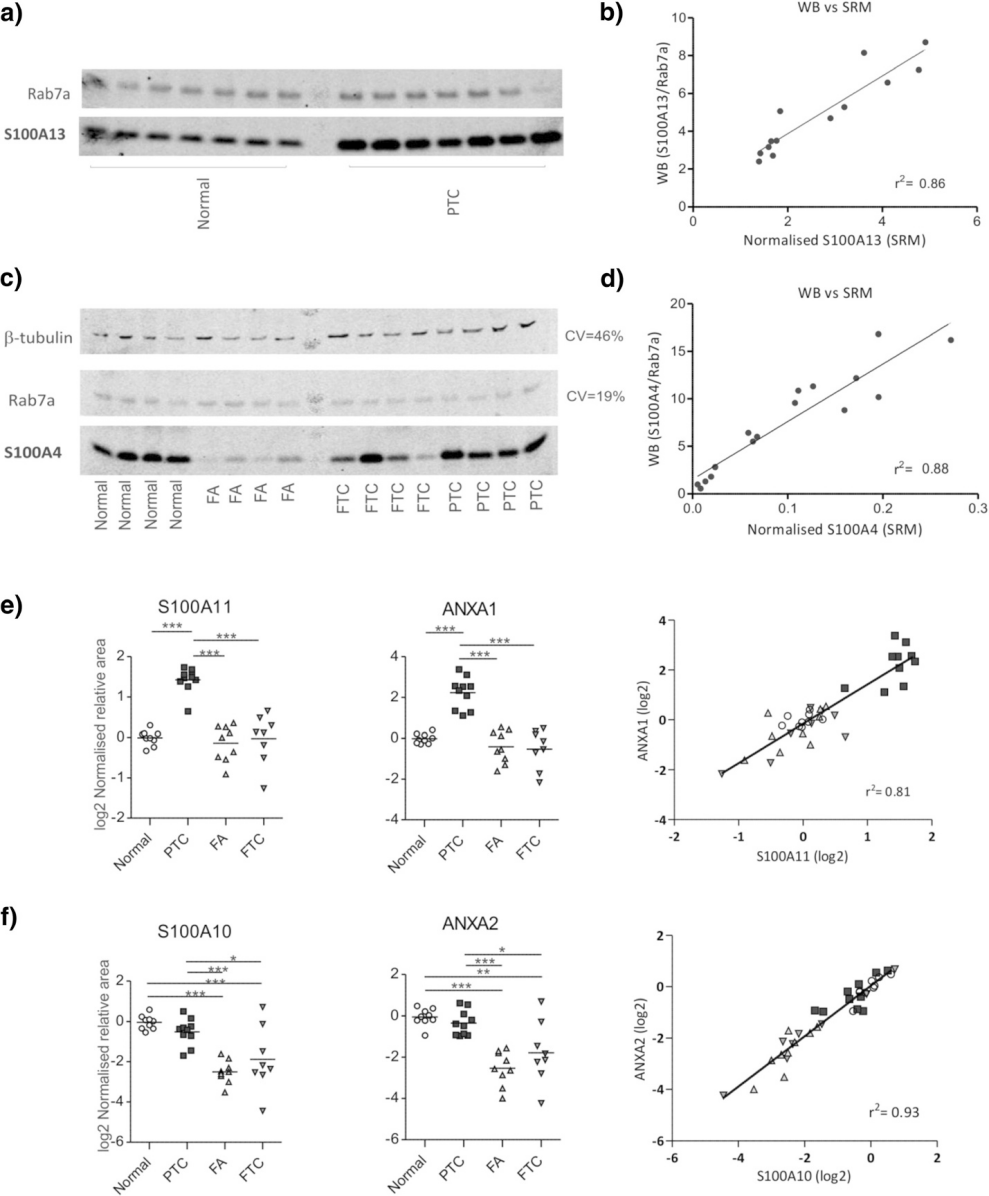
**Validation of SRM-based results and correlated expression of S100 proteins with ANXA1 and ANXA2. a)** Western blot of S100A13 showing overexpression in PTC tissues; **b)** Western Blot of S100A13 (S100A13/Rab7a, one PTC outlier excluded) showed agreement with SRM-based results; **c)** Western blot of S100A4, β-tubulin and Rab7a in thyroid tumours and normal thyroid . β-tubulin presented higher variation (CV = 46%) than Rab7a (CV = 19%); **d)** Normalised S100A4 expression (S100A4/Rab7a) was well correlated with that from SRM analysis. **e)** Expression of S100A11 and its interaction partner ANXA1 are highly correlated across all samples, and are overexpressed in PTC; **f)** Expression of S100A10 and its interaction partner ANXA2 are also highly correlated across all samples, and are both reduced in FA and FTC.

As shown in Figure 2, S100A13 was found overexpressed in tumours of PTC origin (log_2_fc = 1.03, p < 0.05) compared to normal thyroid. Additionally, increased expression of S100A13 was generally observed in FA and FTC specimens, although this varied considerably amongst these samples. Analysis of four publicly available gene expression datasets, three of them comparing matched normal-PTC tumour samples, confirmed the overexpression of S100A13 mRNA transcripts in PTC (Additional file 1: Figure S2). The SRM-based results were validated by Western Blot (Figure 3a) and were highly correlated (r^2^ = 0.86) (Figure 3b).

### S100A4 is frequently reduced in tumours of follicular origin

S100A4 expression was greatly decreased in benign follicular adenomas compared with normal thyroid tissue. FTCs expressed higher levels of S100A4 than FAs, but still less than normal thyroid and PTC specimens (Figure 2). Western Blot confirmed the quantitative MS results of S100A4 expression in the tissue samples (Figure 3c and d).

### S100A6, S100A11 and ANXA1 are markedly overexpressed in PTC and can discriminate tumours of papillary and follicular histology

S100A6 and S100A11 were more abundant in PTCs compared to normal thyroid (log2fc = 1.39 and log2fc = 1.44, respectively), as previously reported [57,58]. ANXA1 is a putative S100A11 interaction partner [59] that we found to be highly overexpressed in PTC (log_2_fc = 2.25) and well correlated with S100A11 expression (r^2^: 0.81, Figure 3e). AUC values after ROC curve analysis demonstrated the high discriminatory power of each of these proteins to distinguish PTC from FA + FTC tissues in this cohort: S100A6 AUC = 0.99, S100A11 AUC = 0.99, ANXA1 AUC = 1.

### FA and FTC show reduced levels of S100A10 and ANXA2

Significantly decreased abundance of S100A10 and its known binding partner ANXA2 [60] was shown for FA (log_2_fc = - 2.47) and FTC (log_2_fc = -1.84) when compared against normal thyroid samples. A similar result was obtained with reference to PTC (Figure 3f). The expression of ANXA2 and S100A10 was highly correlated in all samples (r^2^: 0.93, Figure 3f).

### Other S100 protein isoforms

No significant expression differences were observed for S100A1, S100A2, S100A8, S100A9, S100A14 and S100A16 in FA, FTC and PTC specimens with respect to normal thyroid tissue, except for S100A2 and S100A8 in FA. S100A8 and S100A9 are known to form heterodimers and their coordinated expression was observed in all tissues examined (Additional file 1: Figure S3).

### Results based on label-free SRM of S100 proteins

**Figure 4:**
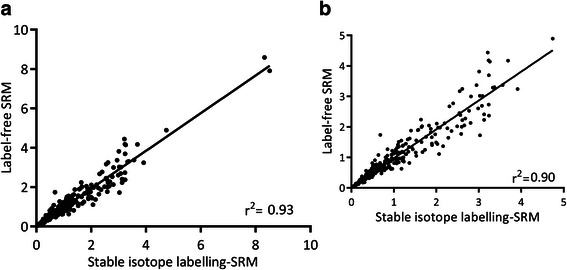
**Label-free analysis of S100 proteins is well correlated with labelling-based quantitation: a) Global comparison between label-based and label-free SRM data of S100 proteins in thyroid tissues (relative values against normal tissues); b) Comparison removing two upper extreme values.**

**Figure 5:**
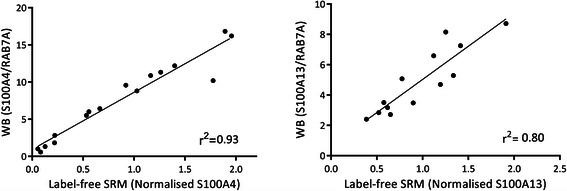
**Comparison of label-free SRM and Western blot analysis of S100A4 and S100A13.** (S100A4, r^2^ 0.93; S100A13, r^2^ 0.80).

The analysis of S100 proteins by label-free SRM provided essentially the same results as those found by stable-isotope labelling (see Figure 4 and Additional file 1: Figure S4). The median CV was higher, 19.9%, which is in line with the results obtained in the analysis of cell lines in our previous work [45] and with other studies by label-free SRM using a spiked standard [61]. S100A1 was not detected in the TPC1 cell line used for SILAC spike-in quantitation and it was analysed using the label-free approach only. Figure 5 shows that label-free SRM analyses are also well correlated with Western blot results, as in the case of SRM with stable-isotope labelling (Figure 3b and d)

## Discussion

The S100 protein family represents a promising panel of biomarkers for cancer and other diseases. However, methods are lacking for the quantitative screening of multiple S100 protein isoforms in clinical specimens. Here, we have showed that targeted MS using SRM enables the specific and multiplexed quantitation of S100 proteins in a large-scale study with 37 tissues of thyroid origin. As our method provided relative quantitation between samples it was necessary to investigate which proteins in thyroid tissues could act as stable endogenous reference proteins, which in this case were Rab7a, cyclophilin A and PSMB2. To the best of our knowledge, this is the first study to identify these loading control proteins for thyroid cancer specimens. Of note, others have already detected dysregulation of β-actin and tubulin isoforms in FA, FTC and PTC [62], hence supporting our strategy. Additionally, Western blot corroborated the variation in expression levels of β-tubulin and Rab7a across different tissue samples (CV of 46% vs. 19%, respectively, Figure 3c).

The MS method harnesses the high accuracy and precision of SILAC-based MS quantitation and expands the number of targeted S100 protein isoforms through the use of label-free SRM. Although the quantitation with the SILAC spike-in achieved greater precision than the label-free experiments, both approaches identified the same significant changes in the tissue samples and were well correlated. Isotope-labelled peptide standards offer an advantage in that LC retention time and non-specific peptide interferences can be readily determined. Moreover, the SRM technique can readily be configured to measure protein concentration through the inclusion of quantified stable isotope peptide standards [63]. While we have determined the appropriate normalisation factors for relative quantitation of S100 proteins in thyroid samples, to extend this to other cancer types we recommend using the SILAC spike-in standard to establish these factors. The full accompaniment of S100 proteins can then be quantitated by label-free SRM of SDS-PAGE fractionated samples, which we have shown to be an effective approach to minimise sample matrix background [45].

Our experiments revealed the novel observation of increased S100A13 in PTC compared with FA, FTC and normal thyroid tissue, and this was supported by Western blot and previous studies of mRNA transcript levels. S100A13 gene silencing in lung cancer cells has been observed to decrease the invasive potential in vitro [64]. Moreover, S100A13 is known to participate in a multi-protein complex that facilitates the release of fibroblast growth factor 1 (FGF-1) [65]. Increased FGF-1 expression has been previously observed in differentiated thyroid cancer (DTC) [66]. Binding of secreted FGFs with heparan sulphate proteoglycans enables signalling through receptor tyrosine kinases to activate MAPK and PI3K/AKT pathways which contribute to tumour growth and angiogenesis [67]. Interestingly, S100A13 upregulation has recently been linked to resistance to the chemotherapeutic agents dacarbazine or temozolomide in melanoma patients [68]. Others have shown S100A13 to be an angiogenic marker [32]. Extrapolating these findings to thyroid cancer implicates S100A13 as a new oncogenic factor in PTC, and this could warrant evaluation of the effectiveness of inhibiting S100A13-driven release of FGF-1.

Overexpression of S100A6 in PTC has been observed previously [57], but more studies are needed to understand its functional significance. S100A11 has recently been shown to reduce the loss of contact inhibition, anchorage-independent growth and resistance to anoikis in PTC cells and enhance the activity of PTC-associated oncogenes such as BRAFV600E and TRK-T3 [58]. We have shown that S100A11 expression is consistent among normal and follicular histotypes, despite being elevated in PTC. The MS method demonstrated that S100A6, S100A11 and ANXA1 differentiate PTC from follicular tumours and that ANXA1 is highly correlated with S100A11 expression. While not directly analysed here, the utility of our findings could be explored for distinguishing the follicular variant of PTC.

FA and FTC cannot be readily distinguished by cytologic evaluation of FNA biopsies, which poses an important diagnostic issue. Our findings using the MS assay show the loss of S100A4 in follicular adenoma tissue compared with follicular thyroid carcinoma, so this difference in expression has diagnostic potential. A previous study did not report the decrease of S100A4 in FA [69]. Our result might be explained by considering that tumour tissue analysis by MS allows the inclusion of both cancer and stromal S100A4-positive cells, which are known to cooperate to drive tumour progression and metastasis [70]. Since S100A4 has well-established pro-migratory, invasive and angiogenic activities [71], it would be of interest to examine S100A4 expression in metastatic radioactive iodine-refractory DTC, where it could emerge as a possible therapeutic target. A proof of concept has already been demonstrated in a mouse model with anaplastic thyroid carcinoma, showing absence of metastasis in mice inoculated with S100A4-shRNA knockdown cells [72].

The reduced expression of S100A10 in FA and FTC was well correlated with the expression of its interaction partner ANXA2. The S100A10-ANXA2 complex anchors to the plasma membrane via the ANXA2 subunits and has an active role in the trafficking of ion channels [60]. A previous IHC study showed only low S100A10 immunoreactivity in PTC tissues while essentially all normal, FA and FTC samples were S100A10-negative [73]. Our results show clearly higher levels of S100A10-ANXA2 in normal tissues compared with FA and FTC. Whether or not loss of S100A10 in the tumour microenvironment exerts a functional role in follicular thyroid neoplasia is a subject of future investigations.

## Conclusions

We have demonstrated the use of targeted mass spectrometry for the multiplexed and specific analysis of S100 protein isoforms in thyroid tumour specimens. This approach provides unambiguous structural evidence of S100 protein isoform expression thereby overcoming potential problems of antigen cross-reactivity that can interfere with affinity detection methods. This study led to the global characterisation of S100 protein expression in thyroid tumours and highlighted several S100 protein isoforms for potential diagnostic and therapeutic evaluation. S100A13 emerged as a novel candidate PTC biomarker. Our results indicate that S100A6, S100A11 and ANXA1 have utility to discriminate follicular and papillary thyroid tumours. The diagnostic and functional significance of S100A4 and S100A10 reduction in follicular tumours warrants further investigations.

## Additional file

Additional file 1: Table S1. Types of cancer where the altered expression of S100 proteins/genes has been observed. For more details, readers are encouraged to consult the reviews [1-5] and literature focused on each S100 protein/cancer. **Table S2.** List of precursors (Q1) and fragment ions (Q3) for detection of S100 peptides. CE: Collision energy, CEXP: Cell exit potential. **Table S3.** Targeted peptide sequences of candidate reference proteins in the region 14-32 kDa. **Table S4.** Targeted peptide sequences of candidate reference proteins in the region 32-100 kDa. **Figure S1.** Expression of S100 proteins S100A1, S100A14 and S100A16 in normal and tumour thyroid tissues as analysed by the MS assay. Normalised expression values (against reference proteins) were log-transformed and significant changes of S100 protein expression were identified by one-way anova followed by Tukey’s post hoc test. Results are expressed against the mean S100 protein expression in normal tissue samples; *p<0.05, **p<0.01, ***p<0.001. **Figure S2.** S100A13 mRNA expression in matched normal-tumour samples from GEO Series a) GSE3467 b) GSE33630 and c) GSE3678. Samples in d) GSE9115 are not matched. Groups were analysed by unpaired (GSE9115) or paired t test (matched samples); *p<0.05, **p<0.01, ***p<0.001. **Figure S3.** S100A8 and S100A9 expression showed good correlation in the normal and tumour tissues. The graph was plotted with log2 values of SRM-based normalised S100 protein expression. **Figure S4.** Relative expression of S100 proteins in thyroid tissues according to label-free SRM data **Figure S4.** (cont). Relative expression of S100 proteins in thyroid tissues according to label-free SRM data.

## Abbreviations

DTC: Differentiated thyroid cancer
FA: Follicular adenoma
Fc: Fold change
FGF-1: Fibroblast growth factor-1
FTC: Follicular thyroid carcinoma
IHC: Immunohistochemistry
MS: Mass spectrometry
SRM: Selected reaction monitoring
PTC: Papillary thyroid carcinoma
