# Supplementary material for: A multiplexed, targeted mass spectrometry assay of the S100 protein family uncovers the isoform-specific expression in thyroid tumours
Source: BMC Cancer. 2015 Mar 29;15:199. doi: 10.1186/s12885-015-1217-x (PMC4391164; doi:10.1186/s12885-015-1217-x)
Supplement: Additional file 1: Table S1. — Types of cancer where the altered expression of S100 proteins/genes has been observed. For more details, readers are encouraged to consult the reviews [1-5] and literature focused on each S100 protein/cancer. Table S2. List of precursors (Q1) and fragment ions (Q3) for detection of S100 peptides. CE: Collision energy, CEXP: Cell exit potential. Table S3. Targeted peptide sequences of candidate reference proteins in the region 14-32 kDa. Table S4. Targeted peptide sequences of candidate reference proteins in the region 32-100 kDa. Figure S1. Expression of S100 proteins S100A1, S100A14 and S100A16 in normal and tumour thyroid tissues as analysed by the MS assay. Normalised expression values (against reference proteins) were log-transformed and significant changes of S100 protein expression were identified by one-way anova followed by Tukey’s post hoc test. Results are expressed against the mean S100 protein expression in normal tissue samples; *p<0.05, **p<0.01, ***p<0.001. Figure S2. S100A13 mRNA expression in matched normal-tumour samples from GEO Series a) GSE3467 b) GSE33630 and c) GSE3678. Samples in d) GSE9115 are not matched. Groups were analysed by unpaired (GSE9115) or paired t test (matched samples); *p<0.05, **p<0.01, ***p<0.001. Figure S3. S100A8 and S100A9 expression showed good correlation in the normal and tumour tissues. The graph was plotted with log2 values of SRM-based normalised S100 protein expression. Figure S4. Relative expression of S100 proteins in thyroid tissues according to label-free SRM data Figure S4. (cont). Relative expression of S100 proteins in thyroid tissues according to label-free SRM data. [file 12885_2015_1217_MOESM1_ESM.docx]

**Additional file 1**

**Table S1**. Types of cancer where the altered expression of S100 proteins/genes has been observed. For more details, readers are encouraged to consult the reviews [1-5] and literature focused on each S100 protein/cancer

| Protein | Cancer |
| --- | --- |
| S100A2 | Breast , oesophageal SCC, gastric, HNSCC, lung, oral, ovarian, prostate, thyroid |
| S100A3 | Colon, gastric |
| S100A4 | Bladder, breast, colorectal, gastric, lung, pancreas, prostate, thyroid |
| S100A6 | Breast, colorectal, gastric, melanoma, NSCLC, pancreas, prostate, thyroid |
| S100A7 | Bladder SCC, breast, oesophageal SCC, lung SCC |
| S100A8/A9 | Bladder, breast, colorectal, gastric, lung, ovarian, pancreas, prostate |
| S100A10 | Thyroid |
| S100A11 | Bladder, breast, colorectal, gastric, NSCLC, pancreatic, prostate, thyroid |
| S100A12 | Colorectal |
| S100A13 | Melanoma |
| S100A14 | Breast, Oesophageal SCC |
| S100B | Melanoma |
| S100P | Breast, colorectal, gastric, hepatocellular, NSCLC, pancreas, prostate |

HNSCC: head and neck squamous cell carcinoma; NSCLC: non-small cell lung cancer; SCC: squamous cell carcinoma

**Table S2.** List of precursors (Q1) and fragment ions (Q3) for detection of S100 peptides. CE: Collision energy, CEXP: Cell exit potential

| Q1 | Q3 | Dwell time (ms) | Protein.peptide.transition ion | CE | CEXP |
| --- | --- | --- | --- | --- | --- |
| 846.45 | 778.41 | 15 | S100A1.ELLQTELSGFLDAQK.+2y7 | 39.3 | 13 |
| 846.45 | 865.44 | 15 | S100A1.ELLQTELSGFLDAQK.+2y8 | 39.3 | 13 |
| 846.45 | 1208.62 | 15 | S100A1.ELLQTELSGFLDAQK.+2y11 | 39.3 | 13 |
| 503.27 | 763.40 | 15 | S100A2.ELPSFVGEK.+2y7 | 22.7 | 12 |
| 503.27 | 666.35 | 15 | S100A2.ELPSFVGEK.+2y6 | 30.7 | 11 |
| 503.27 | 579.31 | 15 | S100A2.ELPSFVGEK.+2y5 | 30.7 | 9 |
| 675.84 | 750.38 | 15 | S100A3.ELATWTPTEFR.+2y6 | 33.2 | 13 |
| 675.84 | 649.33 | 15 | S100A3.ELATWTPTEFR.+2y5 | 33.2 | 13 |
| 675.84 | 936.46 | 15 | S100A3.ELATWTPTEFR.+2y7 | 33.2 | 13 |
| 445.75 | 648.37 | 15 | S100A4.ELPSFLGK.+2y6 | 21.5 | 19 |
| 445.75 | 551.32 | 15 | S100A4.ELPSFLGK.+2y5 | 25.5 | 19 |
| 445.75 | 464.29 | 15 | S100A4.ELPSFLGK.+2y4 | 25.5 | 16 |
| 455.22 | 693.36 | 15 | S100A4.TDEAAFQK.+2y6 | 19.2 | 20 |
| 455.22 | 564.31 | 15 | S100A4.TDEAAFQK.+2y5 | 19.2 | 20 |
| 455.22 | 493.28 | 15 | S100A4.TDEAAFQK.+2y4 | 19.2 | 14 |
| 548.25 | 779.39 | 15 | S100A5.NSDQEIDFK.+2y6 | 28.6 | 13 |
| 548.25 | 651.34 | 15 | S100A5.NSDQEIDFK.+2y7 | 28.6 | 13 |
| 548.25 | 894.42 | 15 | S100A5.NSDQEIDFK.+2y4 | 28.6 | 13 |
| 374.22 | 618.38 | 15 | S100A6.ELTIGSK.+2y6 | 20.3 | 13 |
| 374.22 | 505.30 | 15 | S100A6.ELTIGSK.+2y5 | 20.3 | 13 |
| 374.22 | 404.25 | 15 | S100A6.ELTIGSK.+2y4 | 20.3 | 13 |
| 458.25 | 802.41 | 15 | S100A6.LQDAEIAR.+2y7 | 25.4 | 13 |
| 458.25 | 674.35 | 15 | S100A6.LQDAEIAR.+2y6 | 23.4 | 20 |
| 458.25 | 559.32 | 15 | S100A6.LQDAEIAR.+2y5 | 31.4 | 14 |
| 628.81 | 708.36 | 15 | S100A7.GTNYLADVFEK.+2y6 | 31.5 | 13 |
| 628.81 | 821.44 | 15 | S100A7.GTNYLADVFEK.+2y7 | 31.5 | 13 |
| 628.81 | 984.50 | 15 | S100A7.GTNYLADVFEK.+2y8 | 31.5 | 13 |
| 728.70 | 978.49 | 15 | S100A7.IDFSEFLSLLGDIATDYHK.+3y17+2 | 37.2 | 13 |
| 728.70 | 904.96 | 15 | S100A7.IDFSEFLSLLGDIATDYHK.+3y16+2+ | 37.2 | 13 |
| 728.70 | 1036.01 | 15 | S100A7.IDFSEFLSLLGDIATDYHK.+3y18+2 | 37.2 | 13 |
| 718.70 | 781.91 | 15 | S100A7A.IDFSEFLSLLGDIAADYHK.+3y14+2 | 36.7 | 13 |
| 718.70 | 889.95 | 15 | S100A7A.IDFSEFLSLLGDIAADYHK.+3y16+2 | 36.7 | 13 |
| 718.70 | 963.49 | 15 | S100A7A.IDFSEFLSLLGDIAADYHK.+3y17+2 | 36.7 | 13 |
| 426.57 | 423.22 | 15 | S100A7A.GIHYLATVFEK.+3y3 | 20.8 | 13 |
| 426.57 | 522.29 | 15 | S100A7A.GIHYLATVFEK.+3y4 | 20.8 | 13 |
| 426.57 | 623.34 | 15 | S100A7A.GIHYLATVFEK.+3y5 | 20.8 | 13 |
| 721.32 | 526.25 | 15 | S100A7L2.ENFPNFLSGCEK.+2y9+2 | 34.8 | 13 |
| 721.32 | 693.32 | 15 | S100A7L2.ENFPNFLSGCEK.+2y6 | 34.8 | 13 |
| 721.32 | 1051.49 | 15 | S100A7L2.ENFPNFLSGCEK.+2y9 | 34.8 | 13 |
| 449.19 | 606.25 | 15 | S100A7L2.QYSGDDGR.+2y6 | 25.0 | 13 |
| 449.19 | 519.22 | 15 | S100A7L2.QYSGDDGR.+2y5 | 25.0 | 13 |
| 449.19 | 462.19 | 15 | S100A7L2.QYSGDDGR.+2y4 | 25.0 | 13 |
| 551.04 | 674.87 | 15 | S100A7L2.VNYSEFLSLLGDITIDHHK.+4y12+2 | 27.6 | 13 |
| 551.04 | 574.81 | 15 | S100A7L2.VNYSEFLSLLGDITIDHHK.+4y10+2 | 27.6 | 13 |
| 551.04 | 518.27 | 15 | S100A7L2.VNYSEFLSLLGDITIDHHK.+4y9+2 | 27.6 | 13 |
| 636.85 | 774.41 | 15 | S100A8.ALNSIIDVYHK.+2y6 | 31.8 | 13 |
| 636.85 | 974.53 | 15 | S100A8.ALNSIIDVYHK.+2y8 | 31.8 | 13 |
| 636.85 | 887.50 | 15 | S100A8.ALNSIIDVYHK.+2y7 | 31.8 | 13 |
| 711.36 | 965.45 | 15 | S100A8.LLETECPQYIR.+2y7 | 34.5 | 13 |
| 711.36 | 1066.50 | 15 | S100A8.LLETECPQYIR.+2y8 | 34.5 | 13 |
| 711.36 | 1196.53 | 15 | S100A8.LLETECPQYIR.+2y9 | 34.5 | 13 |
| 411.71 | 694.36 | 15 | S100A8.GADVWFK.+2y5 | 23.7 | 13 |
| 411.71 | 480.26 | 15 | S100A8.GADVWFK.+2y3 | 23.7 | 13 |
| 411.71 | 579.33 | 15 | S100A8.GADVWFK.+2y4 | 23.7 | 13 |
| 602.98 | 790.42 | 15 | S100A9.NIETIINTFHQYSVK.+3y13+2 | 30.4 | 13 |
| 602.98 | 725.89 | 15 | S100A9.NIETIINTFHQYSVK.+3y12+2 | 30.4 | 13 |
| 602.98 | 618.83 | 15 | S100A9.NIETIINTFHQYSVK.+3y10+2 | 30.4 | 13 |
| 728.37 | 1148.56 | 15 | S100A9.LGHPDTLNQGEFK.+2y10 | 35.1 | 13 |
| 728.37 | 1051.51 | 15 | S100A9.LGHPDTLNQGEFK.+2y9 | 35.1 | 13 |
| 728.37 | 936.48 | 15 | S100A9.LGHPDTLNQGEFK.+2y8 | 35.1 | 13 |
| 439.24 | 649.37 | 15 | S100A9.DLQNFLK.+2y5 | 24.7 | 13 |
| 439.24 | 521.31 | 15 | S100A9.DLQNFLK.+2y4 | 24.7 | 13 |
| 439.24 | 407.27 | 15 | S100A9.DLQNFLK.+2y3 | 24.7 | 13 |
| 379.21 | 642.38 | 15 | S100A10.DPLAVDK.+2y6 | 22.5 | 19 |
| 379.21 | 545.33 | 15 | S100A10.DPLAVDK.+2y5 | 20.5 | 17 |
| 379.21 | 432.25 | 15 | S100A10.DPLAVDK.+2y4 | 20.5 | 17 |
| 604.80 | 932.48 | 15 | S100A10.EFPGFLENQK.+2y8 | 26.6 | 13 |
| 604.80 | 835.43 | 15 | S100A10.EFPGFLENQK.+2y7 | 34.6 | 13 |
| 604.80 | 466.75 | 15 | S100A10.EFPGFLENQK.+2y8+2 | 26.6 | 13 |
| 386.20 | 559.32 | 15 | S100A11.DPGVLDR.+2y5 | 24.2 | 18 |
| 386.20 | 502.30 | 15 | S100A11.DPGVLDR.+2y4 | 24.2 | 18 |
| 386.20 | 403.23 | 15 | S100A11.DPGVLDR.+2y3 | 20.2 | 18 |
| 530.75 | 888.45 | 15 | S100A11.DGYNYTLSK.+2y7 | 24.0 | 14 |
| 530.75 | 725.38 | 15 | S100A11.DGYNYTLSK.+2y6 | 24.0 | 12 |
| 530.75 | 611.34 | 15 | S100A11.DGYNYTLSK.+2y5 | 24.0 | 18 |
| 394.73 | 659.41 | 15 | S100A12.ELANTIK.+2y6 | 23.1 | 13 |
| 394.73 | 475.29 | 15 | S100A12.ELANTIK.+2y4 | 23.1 | 13 |
| 394.73 | 546.33 | 15 | S100A12.ELANTIK.+2y5 | 23.1 | 13 |
| 452.73 | 448.28 | 15 | S100A12.GHFDTLSK.+2y4 | 25.2 | 13 |
| 452.73 | 563.30 | 15 | S100A12.GHFDTLSK.+2y5 | 25.2 | 13 |
| 452.73 | 710.37 | 15 | S100A12.GHFDTLSK.+2y6 | 25.2 | 13 |
| 728.38 | 781.41 | 15 | S100A12.LEEHLEGIVNIFHQYSVR.+3y13+2 | 37.2 | 13 |
| 728.38 | 837.95 | 15 | S100A12.LEEHLEGIVNIFHQYSVR.+3y14+2 | 37.2 | 13 |
| 728.38 | 906.48 | 15 | S100A12.LEEHLEGIVNIFHQYSVR.+3y15+2 | 37.2 | 13 |
| 519.76 | 836.45 | 15 | S100A13.DSLSVNEFK.+2y7 | 23.6 | 13 |
| 519.76 | 723.37 | 15 | S100A13.DSLSVNEFK.+2y6 | 23.6 | 20 |
| 519.76 | 636.34 | 15 | S100A13.DSLSVNEFK.+2y5 | 23.6 | 20 |
| 372.24 | 630.38 | 15 | S100A13.LIGELAK.+2y6 | 19.5 | 19 |
| 372.24 | 517.30 | 15 | S100A13.LIGELAK.+2y5 | 17.5 | 14 |
| 372.24 | 460.28 | 15 | S100A13.LIGELAK.+2y4 | 25.5 | 16 |
| 624.31 | 1047.50 | 15 | S100A13.SLDVNQDSELK.+2y9 | 31.3 | 16 |
| 624.31 | 932.47 | 15 | S100A13.SLDVNQDSELK.+2y8 | 31.3 | 16 |
| 624.31 | 833.40 | 15 | S100A13.SLDVNQDSELK.+2y7 | 31.3 | 14 |
| 589.78 | 994.43 | 15 | S100A14.IANLGSCNDSK.+2y9 | 30.1 | 13 |
| 589.78 | 880.38 | 15 | S100A14.IANLGSCNDSK.+2y8 | 30.1 | 13 |
| 589.78 | 767.30 | 15 | S100A14.IANLGSCNDSK.+2y7 | 30.1 | 13 |
| 625.82 | 1016.54 | 15 | S100A14.SFWELIGEAAK.+2y9 | 29.0 | 17 |
| 625.82 | 830.46 | 15 | S100A14.SFWELIGEAAK.+2y8 | 29.0 | 13 |
| 625.82 | 701.42 | 15 | S100A14.SFWELIGEAAK.+2y7 | 32.0 | 12 |
| 834.36 | 1009.46 | 15 | S100A14.SANAEDAQEFSDVER.+2y8 | 38.9 | 13 |
| 834.36 | 881.40 | 15 | S100A14.SANAEDAQEFSDVER.+2y7 | 38.9 | 13 |
| 834.36 | 752.36 | 15 | S100A14.SANAEDAQEFSDVER.+2y6 | 38.9 | 13 |
| 647.87 | 912.48 | 15 | S100A16.AVIVLVENFYK.+2y7 | 28.0 | 14 |
| 647.87 | 799.40 | 15 | S100A16.AVIVLVENFYK.+2y6 | 24.0 | 13 |
| 647.87 | 700.33 | 15 | S100A16.AVIVLVENFYK.+2y5 | 26.0 | 12 |
| 683.35 | 1011.46 | 15 | S100A16.LIQNLDANHDGR.+2y9 | 39.5 | 13 |
| 683.35 | 897.42 | 15 | S100A16.LIQNLDANHDGR.+2y8 | 39.5 | 13 |
| 683.35 | 784.33 | 15 | S100A16.LIQNLDANHDGR.+2y7 | 39.5 | 13 |
| 538.29 | 833.45 | 15 | S100P.ELPGFLQSGK.+2y8 | 24.6 | 13 |
| 538.29 | 736.40 | 15 | S100P.ELPGFLQSGK.+2y7 | 32.6 | 12 |
| 538.29 | 679.38 | 15 | S100P.ELPGFLQSGK.+2y6 | 32.6 | 11 |
| 679.83 | 1108.55 | 15 | S100P.YSGSEGSTQTLTK.+2y11 | 35.3 | 17 |
| 679.83 | 964.49 | 15 | S100P.YSGSEGSTQTLTK.+2y9 | 35.3 | 16 |
| 679.83 | 835.45 | 15 | S100P.YSGSEGSTQTLTK.+2y8 | 35.3 | 13 |
| 802.89 | 1007.50 | 15 | S100G.GPNTLDDLFQELDK.+2y8 | 37.8 | 13 |
| 802.89 | 892.48 | 15 | S100G.GPNTLDDLFQELDK.+2y7 | 37.8 | 13 |
| 802.89 | 1122.53 | 15 | S100G.GPNTLDDLFQELDK.+2y9 | 37.8 | 13 |
| 686.42 | 557.37 | 15 | S100G.LLIQAEFPSLLK.+2y5 | 33.6 | 13 |
| 686.42 | 904.51 | 15 | S100G.LLIQAEFPSLLK.+2y8 | 33.6 | 13 |
| 686.42 | 1032.57 | 15 | S100G.LLIQAEFPSLLK.+2y9 | 33.6 | 13 |
| 494.73 | 590.31 | 15 | S100G.EGDPDQLSK.+2y5 | 26.7 | 13 |
| 494.73 | 687.37 | 15 | S100G.EGDPDQLSK.+2y6 | 26.7 | 13 |
| 494.73 | 802.39 | 15 | S100G.EGDPDQLSK.+2y7 | 26.7 | 13 |
| 898.94 | 1138.61 | 15 | S100G.NGDGEVSFEEFQVLVK.+2y9 | 41.2 | 13 |
| 898.94 | 991.55 | 15 | S100G.NGDGEVSFEEFQVLVK.+2y8 | 41.2 | 13 |
| 898.94 | 862.50 | 15 | S100G.NGDGEVSFEEFQVLVK.+2y7 | 41.2 | 13 |
| 609.99 | 793.41 | 15 | S100B.ELINNELSHFLEEIK.+3y13+2 | 41.8 | 13 |
| 609.99 | 736.87 | 15 | S100B.ELINNELSHFLEEIK.+3y12+2 | 41.8 | 13 |
| 609.99 | 679.85 | 15 | S100B.ELINNELSHFLEEIK.+3y11+2 | 41.8 | 13 |
| 423.71 | 460.28 | 15 | S100B.EQEVVDK.+2y4 | 24.1 | 13 |
| 423.71 | 589.32 | 15 | S100B.EQEVVDK.+2y5 | 24.1 | 13 |
| 423.71 | 717.38 | 15 | S100B.EQEVVDK.+2y6 | 24.1 | 13 |
| 508.28 | 560.30 | 15 | S100Z.IVQDLDANK.+2y5 | 27.2 | 13 |
| 508.28 | 675.33 | 15 | S100Z.IVQDLDANK.+2y6 | 27.2 | 13 |
| 508.28 | 803.39 | 15 | S100Z.IVQDLDANK.+2y7 | 27.2 | 13 |
| 416.72 | 474.29 | 15 | S100Z.ETQLVDK.+2y4 | 23.9 | 13 |
| 416.72 | 602.35 | 15 | S100Z.ETQLVDK.+2y5 | 23.9 | 13 |
| 416.72 | 703.40 | 15 | S100Z.ETQLVDK.+2y6 | 23.9 | 13 |
| 627.81 | 911.43 | 15 | S100Z.ELTEFLSCQK.+2y7 | 31.5 | 13 |
| 627.81 | 782.39 | 15 | S100Z.ELTEFLSCQK.+2y6 | 31.5 | 13 |
| 627.81 | 1012.48 | 15 | S100Z.ELTEFLSCQK.+2y8 | 31.5 | 13 |
| CRP peptide for label-free analyses: | | | |  |  |
| 568.28 | 919.50 | 20.5 | CRP.ESDTSYVSLK.+2y8.heavy | 27.0 | 13 |
| 568.28 | 703.50 | 20.5 | CRP.ESDTSYVSLK.+2y6.heavy | 27.0 | 13 |
| 568.28 | 616.50 | 20.5 | CRP.ESDTSYVSLK.+2y5.heavy | 27.0 | 13 |

**Table S3**. Targeted peptide sequences of candidate reference proteins in the region 14-32 kDa

| Q1 | Q3 | Rt (min) | Protein.peptide.transition ion.isotopologue | CE | CEXP |
| --- | --- | --- | --- | --- | --- |
| 537.30 | 802.43 | 32.0 | TB2.ATVNLLGEEK.y7.light | 28.2 | 13 |
| 537.30 | 901.50 | 32.0 | TB2.ATVNLLGEEK.y8.light | 28.2 | 13 |
| 537.30 | 688.39 | 32.0 | TB2.ATVNLLGEEK.y6.light | 28.2 | 13 |
| 541.31 | 810.44 | 32.0 | TB2.ATVNLLGEEK.y7.heavy | 28.3 | 13 |
| 541.31 | 909.51 | 32.0 | TB2.ATVNLLGEEK.y8.heavy | 28.3 | 13 |
| 541.31 | 696.40 | 32.0 | TB2.ATVNLLGEEK.y6.heavy | 28.3 | 13 |
| 564.80 | 691.34 | 23.5 | PROS26.QPVLSQTEAR.y6.light | 29.2 | 13 |
| 564.80 | 804.42 | 23.5 | PROS26.QPVLSQTEAR.y7.light | 29.2 | 13 |
| 564.80 | 604.30 | 23.5 | PROS26.QPVLSQTEAR.y5.light | 29.2 | 13 |
| 569.80 | 701.35 | 23.5 | PROS26.QPVLSQTEAR.y6.heavy | 29.4 | 13 |
| 569.80 | 814.43 | 23.5 | PROS26.QPVLSQTEAR.y7.heavy | 29.4 | 13 |
| 569.80 | 614.31 | 23.5 | PROS26.QPVLSQTEAR.y5.heavy | 29.4 | 13 |
| 738.38 | 873.52 | 48.0 | RAB7A.DPENFPFVVLGNK.y8.light | 35.4 | 13 |
| 738.38 | 1020.59 | 48.0 | RAB7A.DPENFPFVVLGNK.y9.light | 35.4 | 13 |
| 738.38 | 1134.63 | 48.0 | RAB7A.DPENFPFVVLGNK.y10.light | 35.4 | 13 |
| 742.39 | 881.53 | 48.0 | RAB7A.DPENFPFVVLGNK.y8.heavy | 35.6 | 13 |
| 742.39 | 1028.60 | 48.0 | RAB7A.DPENFPFVVLGNK.y9.heavy | 35.6 | 13 |
| 742.39 | 1142.64 | 48.0 | RAB7A.DPENFPFVVLGNK.y10.heavy | 35.6 | 13 |
| 529.32 | 845.47 | 34.0 | RAB7A.VIILGDSGVGK.y9.light | 27.9 | 13 |
| 529.32 | 732.39 | 34.0 | RAB7A.VIILGDSGVGK.y8.light | 27.9 | 13 |
| 529.32 | 619.30 | 34.0 | RAB7A.VIILGDSGVGK.y7.light | 27.9 | 13 |
| 533.33 | 853.49 | 34.0 | RAB7A.VIILGDSGVGK.y9.heavy | 28.1 | 13 |
| 533.33 | 740.40 | 34.0 | RAB7A.VIILGDSGVGK.y8.heavy | 28.1 | 13 |
| 533.33 | 627.32 | 34.0 | RAB7A.VIILGDSGVGK.y7.heavy | 28.1 | 13 |
| 806.39 | 948.49 | 37.0 | PSMB2.NGYELSPTAAANFTR.y9.light | 37.9 | 13 |
| 806.39 | 1035.52 | 37.0 | PSMB2.NGYELSPTAAANFTR.y10.light | 37.9 | 13 |
| 806.39 | 1148.61 | 37.0 | PSMB2.NGYELSPTAAANFTR.y11.light | 37.9 | 13 |
| 811.39 | 958.50 | 37.0 | PSMB2.NGYELSPTAAANFTR.y9.heavy | 38.1 | 13 |
| 811.39 | 1045.53 | 37.0 | PSMB2.NGYELSPTAAANFTR.y10.heavy | 38.1 | 13 |
| 811.39 | 1158.61 | 37.0 | PSMB2.NGYELSPTAAANFTR.y11.heavy | 38.1 | 13 |
| 653.88 | 706.39 | 53.0 | PSMB2.FILNLPTFSVR.y6.light | 32.4 | 13 |
| 653.88 | 933.52 | 53.0 | PSMB2.FILNLPTFSVR.y8.light | 32.4 | 13 |
| 653.88 | 1046.60 | 53.0 | PSMB2.FILNLPTFSVR.y9.light | 32.4 | 13 |
| 658.88 | 716.40 | 53.0 | PSMB2.FILNLPTFSVR.y6.heavy | 32.6 | 13 |
| 658.88 | 943.52 | 53.0 | PSMB2.FILNLPTFSVR.y8.heavy | 32.6 | 13 |
| 658.88 | 1056.61 | 53.0 | PSMB2.FILNLPTFSVR.y9.heavy | 32.6 | 13 |
| 577.79 | 878.46 | 39.0 | CyclophilinA.FEDENFILK.y7.light | 29.7 | 13 |
| 577.79 | 763.43 | 39.0 | CyclophilinA.FEDENFILK.y6.light | 29.7 | 13 |
| 577.79 | 634.39 | 39.0 | CyclophilinA.FEDENFILK.y5.light | 29.7 | 13 |
| 581.80 | 886.48 | 39.0 | CyclophilinA.FEDENFILK.y7.heavy | 29.8 | 13 |
| 581.80 | 771.45 | 39.0 | CyclophilinA.FEDENFILK.y6.heavy | 29.8 | 13 |
| 581.80 | 642.41 | 39.0 | CyclophilinA.FEDENFILK.y5.heavy | 29.8 | 13 |
| 528.27 | 869.44 | 43.0 | CyclophilinA.VSFELFADK.y7.light | 27.9 | 13 |
| 528.27 | 956.47 | 43.0 | CyclophilinA.VSFELFADK.y8.light | 27.9 | 13 |
| 528.27 | 722.37 | 43.0 | CyclophilinA.VSFELFADK.y6.light | 27.9 | 13 |
| 532.28 | 877.45 | 43.0 | CyclophilinA.VSFELFADK.y7.heavy | 28.0 | 13 |
| 532.28 | 964.49 | 43.0 | CyclophilinA.VSFELFADK.y8.heavy | 28.0 | 13 |
| 532.28 | 730.39 | 43.0 | CyclophilinA.VSFELFADK.y6.heavy | 28.0 | 13 |
| 546.30 | 619.36 | 30.5 | RPS13.GLSQSALPYR.y5.light | 28.5 | 13 |
| 546.30 | 706.39 | 30.5 | RPS13.GLSQSALPYR.y6.light | 28.5 | 13 |
| 546.30 | 834.45 | 30.5 | RPS13.GLSQSALPYR.y7.light | 28.5 | 13 |
| 551.30 | 629.37 | 30.5 | RPS13.GLSQSALPYR.y5.heavy | 28.7 | 13 |
| 551.30 | 716.40 | 30.5 | RPS13.GLSQSALPYR.y6.heavy | 28.7 | 13 |
| 551.30 | 844.46 | 30.5 | RPS13.GLSQSALPYR.y7.heavy | 28.7 | 13 |
| 627.38 | 670.46 | 43.0 | RPS13.GLTPSQIGVILR.y6.light | 31.4 | 13 |
| 627.38 | 885.55 | 43.0 | RPS13.GLTPSQIGVILR.y8.light | 31.4 | 13 |
| 627.38 | 982.60 | 43.0 | RPS13.GLTPSQIGVILR.y9.light | 31.4 | 13 |
| 632.38 | 680.47 | 43.0 | RPS13.GLTPSQIGVILR.y6.heavy | 31.6 | 13 |
| 632.38 | 895.56 | 43.0 | RPS13.GLTPSQIGVILR.y8.heavy | 31.6 | 13 |
| 632.38 | 992.61 | 43.0 | RPS13.GLTPSQIGVILR.y9.heavy | 31.6 | 13 |
| 525.28 | 886.49 | 36.0 | RPL27.YSVDIPLDK.y8.light | 27.8 | 13 |
| 525.28 | 700.39 | 36.0 | RPL27.YSVDIPLDK.y6.light | 27.8 | 13 |
| 525.28 | 799.46 | 36.0 | RPL27.YSVDIPLDK.y7.light | 27.8 | 13 |
| 529.29 | 894.50 | 36.0 | RPL27.YSVDIPLDK.y8.heavy | 27.9 | 13 |
| 529.29 | 708.40 | 36.0 | RPL27.YSVDIPLDK.y6.heavy | 27.9 | 13 |
| 529.29 | 807.47 | 36.0 | RPL27.YSVDIPLDK.y7.heavy | 27.9 | 13 |
| 413.78 | 416.26 | 35.0 | RPL27.VVLVLAGR.y4.light | 23.8 | 13 |
| 413.78 | 515.33 | 35.0 | RPL27.VVLVLAGR.y5.light | 23.8 | 13 |
| 413.78 | 628.41 | 35.0 | RPL27.VVLVLAGR.y6.light | 23.8 | 13 |
| 418.78 | 426.27 | 35.0 | RPL27.VVLVLAGR.y4.heavy | 23.9 | 13 |
| 418.78 | 525.34 | 35.0 | RPL27.VVLVLAGR.y5.heavy | 23.9 | 13 |
| 418.78 | 638.42 | 35.0 | RPL27.VVLVLAGR.y6.heavy | 23.9 | 13 |
| 585.81 | 625.32 | 38.5 | RPL9.FLDGIYVSEK.y5.light | 29.9 | 13 |
| 585.81 | 795.42 | 38.5 | RPL9.FLDGIYVSEK.y7.light | 29.9 | 13 |
| 585.81 | 910.45 | 38.5 | RPL9.FLDGIYVSEK.y8.light | 29.9 | 13 |
| 589.82 | 633.33 | 38.5 | RPL9.FLDGIYVSEK.y5.heavy | 30.1 | 13 |
| 589.82 | 803.44 | 38.5 | RPL9.FLDGIYVSEK.y7.heavy | 30.1 | 13 |
| 589.82 | 918.47 | 38.5 | RPL9.FLDGIYVSEK.y8.heavy | 30.1 | 13 |
| 621.84 | 830.47 | 32.0 | RPL22.AGNLGGGVVTIER.y8.light | 31.2 | 13 |
| 621.84 | 887.49 | 32.0 | RPL22.AGNLGGGVVTIER.y9.light | 31.2 | 13 |
| 621.84 | 1000.58 | 32.0 | RPL22.AGNLGGGVVTIER.y10.light | 31.2 | 13 |
| 626.84 | 840.48 | 32.0 | RPL22.AGNLGGGVVTIER.y8.heavy | 31.4 | 13 |
| 626.84 | 897.50 | 32.0 | RPL22.AGNLGGGVVTIER.y9.heavy | 31.4 | 13 |
| 626.84 | 1010.59 | 32.0 | RPL22.AGNLGGGVVTIER.y10.heavy | 31.4 | 13 |
| 604.33 | 793.41 | 35.5 | RPL22.ITVTSEVPFSK.y7.light | 30.6 | 13 |
| 604.33 | 894.46 | 35.5 | RPL22.ITVTSEVPFSK.y8.light | 30.6 | 13 |
| 604.33 | 993.53 | 35.5 | RPL22.ITVTSEVPFSK.y9.light | 30.6 | 13 |
| 608.34 | 801.42 | 35.5 | RPL22.ITVTSEVPFSK.y7.heavy | 30.8 | 13 |
| 608.34 | 902.47 | 35.5 | RPL22.ITVTSEVPFSK.y8.heavy | 30.8 | 13 |
| 608.34 | 1001.54 | 35.5 | RPL22.ITVTSEVPFSK.y9.heavy | 30.8 | 13 |
| 594.33 | 705.40 | 38.5 | RPS16.GPLQSVQVFGR.y6.light | 30.3 | 13 |
| 594.33 | 792.44 | 38.5 | RPS16.GPLQSVQVFGR.y7.light | 30.3 | 13 |
| 594.33 | 920.49 | 38.5 | RPS16.GPLQSVQVFGR.y8.light | 30.3 | 13 |
| 599.33 | 715.41 | 38.5 | RPS16.GPLQSVQVFGR.y6.heavy | 30.4 | 13 |
| 599.33 | 802.44 | 38.5 | RPS16.GPLQSVQVFGR.y7.heavy | 30.4 | 13 |
| 599.33 | 930.50 | 38.5 | RPS16.GPLQSVQVFGR.y8.heavy | 30.4 | 13 |
| 547.86 | 739.51 | 49.0 | RPS16.LLEPVLLLGK.y7.light | 28.6 | 13 |
| 547.86 | 868.55 | 49.0 | RPS16.LLEPVLLLGK.y8.light | 28.6 | 13 |
| 547.86 | 981.62 | 49.0 | RPS16.LLEPVLLLGK.y9.light | 28.6 | 13 |
| 551.87 | 747.52 | 49.0 | RPS16.LLEPVLLLGK.y7.heavy | 28.7 | 13 |
| 551.87 | 876.57 | 49.0 | RPS16.LLEPVLLLGK.y8.heavy | 28.7 | 13 |
| 551.87 | 989.63 | 49.0 | RPS16.LLEPVLLLGK.y9.heavy | 28.7 | 13 |
| 533.80 | 668.35 | 27.5 | RPS12.TALIHDGLAR.y6.light | 28.1 | 13 |
| 533.80 | 781.43 | 27.5 | RPS12.TALIHDGLAR.y7.light | 28.1 | 13 |
| 533.80 | 894.52 | 27.5 | RPS12.TALIHDGLAR.y8.light | 28.1 | 13 |
| 538.80 | 678.36 | 27.5 | RPS12.TALIHDGLAR.y6.heavy | 28.3 | 13 |
| 538.80 | 791.44 | 27.5 | RPS12.TALIHDGLAR.y7.heavy | 28.3 | 13 |
| 538.80 | 904.52 | 27.5 | RPS12.TALIHDGLAR.y8.heavy | 28.3 | 13 |
| 567.78 | 678.36 | 27.0 | RPS19.DVNQQEFVR.y5.light | 29.3 | 13 |
| 567.78 | 806.42 | 27.0 | RPS19.DVNQQEFVR.y6.light | 29.3 | 13 |
| 567.78 | 920.46 | 27.0 | RPS19.DVNQQEFVR.y7.light | 29.3 | 13 |
| 572.78 | 688.37 | 27.0 | RPS19.DVNQQEFVR.y5.heavy | 29.3 | 13 |
| 572.78 | 816.42 | 27.0 | RPS19.DVNQQEFVR.y6.heavy | 29.3 | 13 |
| 572.78 | 930.47 | 27.0 | RPS19.DVNQQEFVR.y7.heavy | 29.3 | 13 |
| 415.74 | 561.29 | 27.5 | RPS9.IGVLDEGK.y5.light | 23.8 | 13 |
| 415.74 | 660.36 | 27.5 | RPS9.IGVLDEGK.y6.light | 23.8 | 13 |
| 415.74 | 717.38 | 27.5 | RPS9.IGVLDEGK.y7.light | 23.8 | 13 |
| 419.75 | 569.30 | 27.5 | RPS9.IGVLDEGK.y5.heavy | 23.8 | 13 |
| 419.75 | 668.37 | 27.5 | RPS9.IGVLDEGK.y6.heavy | 23.8 | 13 |
| 419.75 | 725.39 | 27.5 | RPS9.IGVLDEGK.y7.heavy | 23.8 | 13 |

**Table S4**. Targeted peptide sequences of candidate reference proteins in the region 32-100 kDa

| Q1 | Q3 | Rt | Protein.peptide.transition ion.isotopologue | CE | CEXP |
| --- | --- | --- | --- | --- | --- |
| 916.51 | 1191.66 | 80 | GPI.TLAQLNPESSLFIIASK.y11.light | 41.9 | 13 |
| 916.51 | 1094.61 | 80 | GPI.TLAQLNPESSLFIIASK.y10.light | 41.9 | 13 |
| 916.51 | 965.57 | 80 | GPI.TLAQLNPESSLFIIASK.y9.light | 41.9 | 13 |
| 920.52 | 1199.68 | 80 | GPI.TLAQLNPESSLFIIASK.y11.heavy | 42.0 | 13 |
| 920.52 | 1102.62 | 80 | GPI.TLAQLNPESSLFIIASK.y10.heavy | 42.0 | 13 |
| 920.52 | 973.58 | 80 | GPI.TLAQLNPESSLFIIASK.y9.heavy | 42.0 | 13 |
| 534.95 | 659.34 | 55 | GPI.VWYVSNIDGTHIAK.y12.light | 26.7 | 13 |
| 534.95 | 1055.55 | 55 | GPI.VWYVSNIDGTHIAK.y10.light | 26.7 | 13 |
| 534.95 | 741.39 | 55 | GPI.VWYVSNIDGTHIAK.y7.light | 26.7 | 13 |
| 537.62 | 663.35 | 55 | GPI.VWYVSNIDGTHIAK.y12.heavy | 26.8 | 13 |
| 537.62 | 1063.56 | 55 | GPI.VWYVSNIDGTHIAK.y10.heavy | 26.8 | 13 |
| 537.62 | 749.40 | 55 | GPI.VWYVSNIDGTHIAK.y7.heavy | 26.8 | 13 |
| 882.41 | 1101.46 | 68 | GAPDH.LISWYDNEFGYSNR.y9.light | 40.6 | 13 |
| 882.41 | 986.43 | 68 | GAPDH.LISWYDNEFGYSNR.y8.light | 40.6 | 13 |
| 882.41 | 872.39 | 68 | GAPDH.LISWYDNEFGYSNR.y7.light | 40.6 | 13 |
| 887.41 | 1111.47 | 68 | GAPDH.LISWYDNEFGYSNR.y9.heavy | 40.8 | 13 |
| 887.41 | 996.44 | 68 | GAPDH.LISWYDNEFGYSNR.y8.heavy | 40.8 | 13 |
| 887.41 | 882.40 | 68 | GAPDH.LISWYDNEFGYSNR.y7.heavy | 40.8 | 13 |
| 807.45 | 1003.56 | 75 | GAPDH.LVINGNPITIFQER.y8.light | 37.9 | 13 |
| 807.45 | 1117.60 | 75 | GAPDH.LVINGNPITIFQER.y9.light | 37.9 | 13 |
| 807.45 | 1174.62 | 75 | GAPDH.LVINGNPITIFQER.y10.light | 37.9 | 13 |
| 812.45 | 1013.57 | 75 | GAPDH.LVINGNPITIFQER.y8.heavy | 38.1 | 13 |
| 812.45 | 1127.61 | 75 | GAPDH.LVINGNPITIFQER.y9.heavy | 38.1 | 13 |
| 812.45 | 1184.63 | 75 | GAPDH.LVINGNPITIFQER.y10.heavy | 38.1 | 13 |
| 652.03 | 639.38 | 55 | β-Actin.VAPEEHPVLLTEAPLNPK.y6.light | 33.1 | 13 |
| 652.03 | 768.43 | 55 | β-Actin.VAPEEHPVLLTEAPLNPK.y7.light | 33.1 | 13 |
| 652.03 | 869.47 | 55 | β-Actin.VAPEEHPVLLTEAPLNPK.y8.light*** | 33.1 | 13 |
| 654.70 | 647.39 | 55 | β-Actin.VAPEEHPVLLTEAPLNPK.y6.heavy | 33.2 | 13 |
| 654.70 | 776.44 | 55 | β-Actin.VAPEEHPVLLTEAPLNPK.y7.heavy | 33.2 | 13 |
| 654.70 | 877.49 | 55 | β-Actin.VAPEEHPVLLTEAPLNPK.y8.heavy | 33.2 | 13 |
| 651.32 | 839.39 | 42 | β-Tubulin.ISVYYNEATGGK.y8.light | 32.3 | 13 |
| 651.32 | 1002.45 | 42 | β-Tubulin.ISVYYNEATGGK.y9.light | 32.3 | 13 |
| 651.32 | 1188.55 | 42 | β-Tubulin.ISVYYNEATGGK.y11.light | 32.3 | 13 |
| 655.33 | 847.40 | 42 | β-Tubulin.ISVYYNEATGGK.y8.heavy | 32.4 | 13 |
| 655.33 | 1010.47 | 42 | β-Tubulin.ISVYYNEATGGK.y9.heavy | 32.4 | 13 |
| 655.33 | 1196.57 | 42 | β-Tubulin.ISVYYNEATGGK.y11.heavy | 32.4 | 13 |
| 851.95 | 801.48 | 85 | ANXA1.GLGTDEDTLIEILASR.y7.light | 39.5 | 13 |
| 851.95 | 914.57 | 85 | ANXA1.GLGTDEDTLIEILASR.y8.light | 39.5 | 13 |
| 851.95 | 1015.62 | 85 | ANXA1.GLGTDEDTLIEILASR.y9.light | 39.5 | 13 |
| 856.95 | 811.49 | 85 | ANXA1.GLGTDEDTLIEILASR.y7.heavy | 39.7 | 13 |
| 856.95 | 924.58 | 85 | ANXA1.GLGTDEDTLIEILASR.y8.heavy | 39.7 | 13 |
| 856.95 | 1025.62 | 85 | ANXA1.GLGTDEDTLIEILASR.y9.heavy | 39.7 | 13 |
| 786.06 | 927.49 | 59 | ANXA1.GGPGSAVSPYPTFNPSSDVAALHK.y9.light | 40.4 | 13 |
| 786.06 | 1024.54 | 59 | ANXA1.GGPGSAVSPYPTFNPSSDVAALHK.y10.light | 40.4 | 13 |
| 786.06 | 1138.59 | 59 | ANXA1.GGPGSAVSPYPTFNPSSDVAALHK.y11.light | 40.4 | 13 |
| 788.73 | 935.50 | 59 | ANXA1.GGPGSAVSPYPTFNPSSDVAALHK.y9.heavy | 40.4 | 13 |
| 788.73 | 1032.56 | 59 | ANXA1.GGPGSAVSPYPTFNPSSDVAALHK.y10.heavy | 40.4 | 13 |
| 788.73 | 1146.60 | 59 | ANXA1.GGPGSAVSPYPTFNPSSDVAALHK.y11.heavy | 40.4 | 13 |
| 771.93 | 730.42 | 79 | ANXA2.GVDEVTIVNILTNR.y6.light | 36.6 | 13 |
| 771.93 | 829.49 | 79 | ANXA2.GVDEVTIVNILTNR.y7.light | 36.6 | 13 |
| 771.93 | 1043.62 | 79 | ANXA2.GVDEVTIVNILTNR.y9.light | 36.6 | 13 |
| 776.93 | 740.43 | 79 | ANXA2.GVDEVTIVNILTNR.y6.heavy | 36.8 | 13 |
| 776.93 | 839.50 | 79 | ANXA2.GVDEVTIVNILTNR.y7.heavy | 36.8 | 13 |
| 776.93 | 1053.63 | 79 | ANXA2.GVDEVTIVNILTNR.y9.heavy | 36.8 | 13 |
| 906.00 | 813.46 | 97.5 | VCP.NAPAIIFIDELDAIAPK.y15.light | 41.5 | 13 |
| 906.00 | 971.50 | 97.5 | VCP.NAPAIIFIDELDAIAPK.y9.light | 41.5 | 13 |
| 906.00 | 1231.66 | 97.5 | VCP.NAPAIIFIDELDAIAPK.y11.light | 41.5 | 13 |
| 910.01 | 817.47 | 97.5 | VCP.NAPAIIFIDELDAIAPK.y15.heavy | 41.6 | 13 |
| 910.01 | 979.52 | 97.5 | VCP.NAPAIIFIDELDAIAPK.y9.heavy | 41.6 | 13 |
| 910.01 | 1239.67 | 97.5 | VCP.NAPAIIFIDELDAIAPK.y11.heavy | 41.6 | 13 |
| 778.93 | 974.52 | 75 | VCP.LDQLIYIPLPDEK.y8.light | 36.9 | 13 |
| 778.93 | 811.46 | 75 | VCP.LDQLIYIPLPDEK.y7.light | 36.9 | 13 |
| 778.93 | 1087.60 | 75 | VCP.LDQLIYIPLPDEK.y9.light | 36.9 | 13 |
| 782.94 | 982.53 | 75 | VCP.LDQLIYIPLPDEK.y8.heavy | 37.0 | 13 |
| 782.94 | 819.47 | 75 | VCP.LDQLIYIPLPDEK.y7.heavy | 37.0 | 13 |
| 782.94 | 1095.62 | 75 | VCP.LDQLIYIPLPDEK.y9.heavy | 37.0 | 13 |

CE= Collision energy; CEXP= Cell exit potential; ***Actin peptide shared between α and β isoforms

Protein TB2: receptor expression-enhancing protein 5; PROS26: proteasome subunit beta type- 4; RAB7A: Ras-related protein Rab7a; PSMB2: proteasome subunit beta type-2; GPI: glucose-6-phosphate isomerase; VCP: valosin containing protein; Ribosomal proteins: RPS13, RPL27, RPL9, RPL22, RPS16, RPS12, RPS19, RPS9

**Figure S1**. Expression of S100 proteins S100A1, S100A14 and S100A16 in normal and tumour thyroid tissues as analysed by the MS assay. Normalised expression values (against reference proteins) were log-transformed and significant changes of S100 protein expression were identified by one-way anova followed by Tukey’s post hoc test. Results are expressed against the mean S100 protein expression in normal tissue samples; *p<0.05, **p<0.01, ***p<0.001

**Figure S2**. S100A13 mRNA expression in matched normal-tumour samples from GEO Series a) GSE3467 b) GSE33630 and c) GSE3678. Samples in d) GSE9115 are not matched. Groups were analysed by unpaired (GSE9115) or paired t test (matched samples); *p<0.05, **p<0.01, ***p<0.001.

**Figure S3.** S100A8 and S100A9 expression showed good correlation in the normal and tumour tissues. The graph was plotted with log2 values of SRM-based normalised S100 protein expression

**Figure S4.** Relative expression of S100 proteins in thyroid tissues according to label-free SRM data

**Figure S4 (cont).** Relative expression of S100 proteins in thyroid tissues according to label-free SRM data
